# Comparison of Venae Sectio vs. modified Seldinger Technique for Totally Implantable Access Ports; Portas-trial [ISRCTN:52368201]

**DOI:** 10.1186/1745-6215-7-20

**Published:** 2006-06-08

**Authors:** P Knebel, B Fröhlich, H-P Knaebel, P Kienle, S Luntz, MW Buchler, CM Seiler

**Affiliations:** 1 Department of Surgery, University of Heidelberg, Heidelberg, Germany; 2 Coordination Centre for Clinical Trials, University of Heidelberg, Heidelberg, Germany

## Abstract

**Background:**

The insertion of a Totally Implantable Access Port (TIAP) is a routinely employed technique in patients who need a safe and permanent venous access. The number of TIAP implantations is increasing constantly mainly due to advanced treatment options for malignant diseases. Therefore it is important to identify the implantation technique which has the optimal benefit/risk ratio for the patient.

**Study design:**

A single-centre, randomized, controlled superiority trial to compare two different TIAP implantation techniques. Sample size: 160 patients will be included and randomized intra-operatively. Eligibility criteria: Age equal or older than 18 years, patients scheduled for primary elective implantation of a TIAP in local anaesthesia and a signed informed consent. Primary endpoint: Primary success rate of the randomized technique. Intervention: Venae Sectio in combination with the Seldinger Technique (guide wire and a peel away sheath) will be used to place a TIAP. Reference treatment: Conventional Venae Sectio will be used with a direct insertion of the TIAP without guide wire or peel away sheath. Duration of study: Approximately 20 months.

**Organisation/Responsibility:**

The trial will be conducted in compliance with the protocol and in accordance with the moral, ethical, and scientific principles governing clinical research as set out in the Declaration of Helsinki (1989) and Good Clinical Practice (GCP). The trial will also be carried out in keeping with local and regulatory requirements. The Klinisches Studienzentrum Chirurgie (KSC) – Centre of Clinical Trials in Surgery at the Department of Surgery, University Hospital Heidelberg is responsible for planning and conduction of the trial. Documentation of patient's data will be accomplished via electronical Case Report Files (eCRF) with MACRO^®^-Software by the KSC. Randomization, data management, monitoring and biometry are provided by the independent Koordinierungszentrum für Klinische Studien (KKS) – Coordination Centre for Clinical Trails at the University of Heidelberg.

## Background

### Totally Implantable Access Ports (TIAP)

Insertion of a Totally Implantable Access Port (TIAP) is a routinely employed technique in patients who need a save and permanent venous access for e.g. chemotherapy and/or parenteral nutrition[1]. This system needs no external dressing, allows the patient normal physical activity, is probably less prone to infectious complications and will minimize the occlusion rate of the catheter compared to non-totally implantable catheters[1]. TIAPs are being extensively used world-wide and an increase of port placement can be expected with the broader introduction of innovative therapies in oncology such as the Capri Protocol in pancreatic cancer[2].

### Implantation techniques

**Figure 1:**
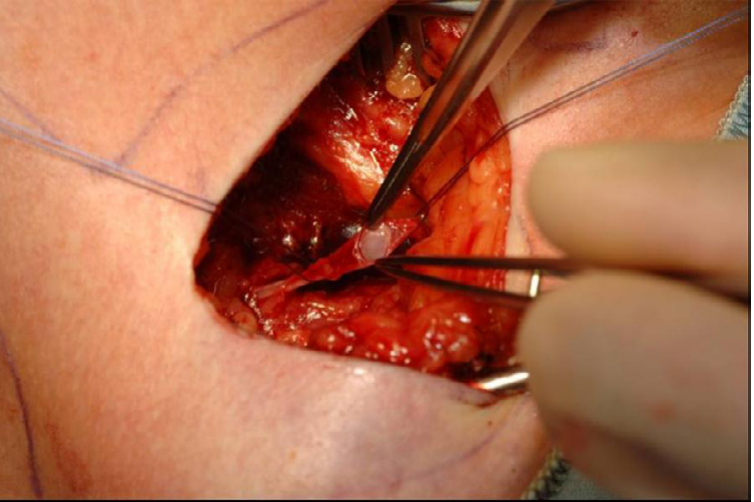
Venae Sectio - Incision of V. cephalica.

**Figure 2:**
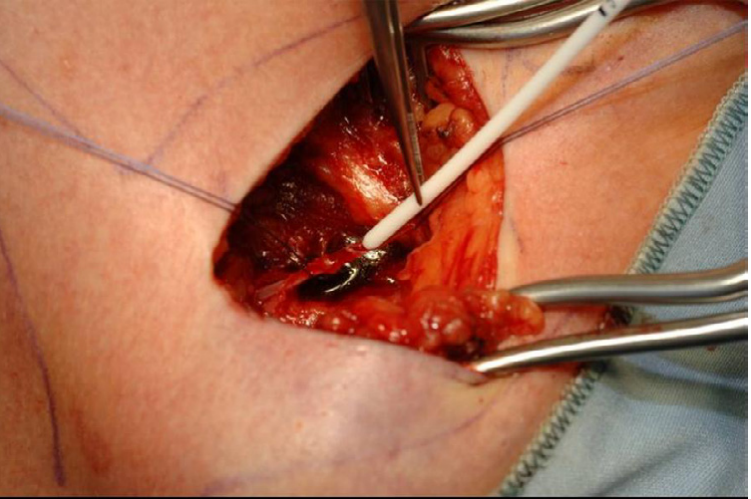
Venae Sectio - Insertion of the catheter.

Until today, two alternative approaches to access the central venous system are in use: Blind puncture of a central vein and introduction of a catheter using the Seldinger Technique or implantation of the TIAP through a surgically dissected vein (e.g. Venae Sectio of the cephalic vein; Fig. 1, 2). Both procedures are currently performed by surgeons as well as interventional radiologists and have a certain risk of complications with respect to the applied technique (pneumothorax, hematothorax, "pinch off" phenomena i.e. kinking of the catheter, nerve palsy, thoracic duct injury)[1,3].

### Modified Seldinger Technique (combination of Venae Sectio and Seldinger Technique)

**Figure 3:**
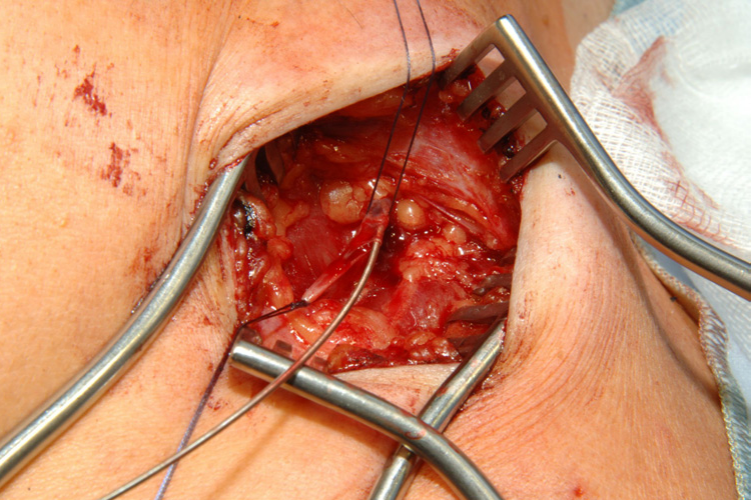
Venae Sectio + Seldinger - Insertion of the guide wire.

**Figure 4:**
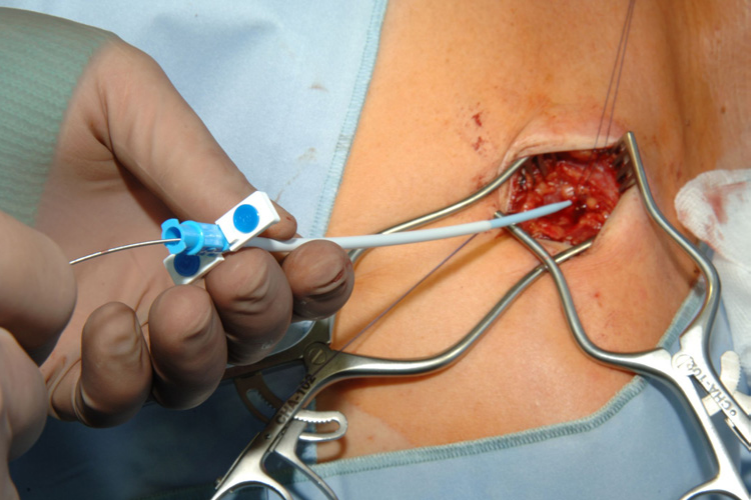
Venae Sectio + Seldinger - Insertion of the dilatator and peel away sheath over the guide wire.

**Figure 5:**
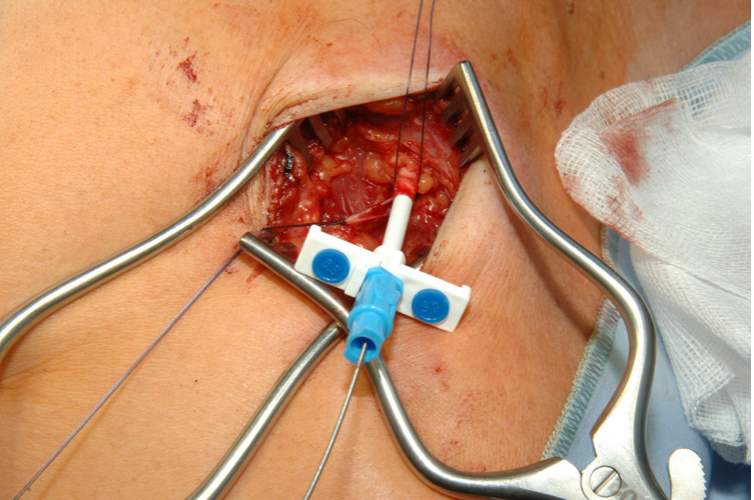
Venae Sectio + Seldinger - Insertion of the dilatator and peel away sheath over the guide wire.

**Figure 6:**
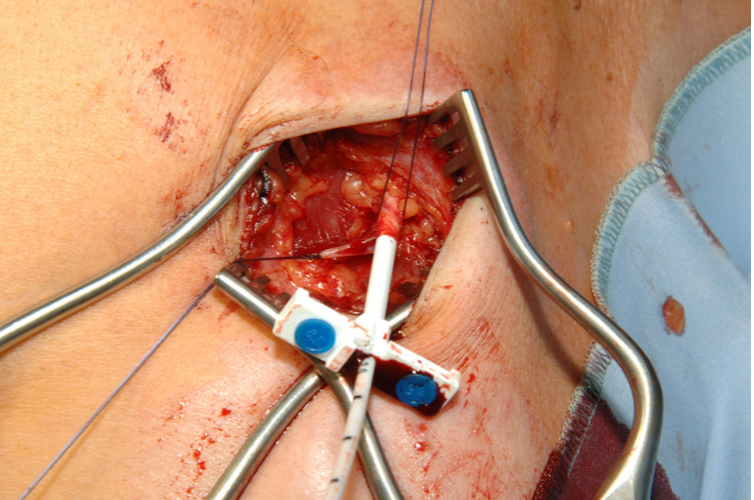
Venae Sectio + Seldinger - Dilatator removed and catheter introduced over the guide wire.

**Figure 7:**
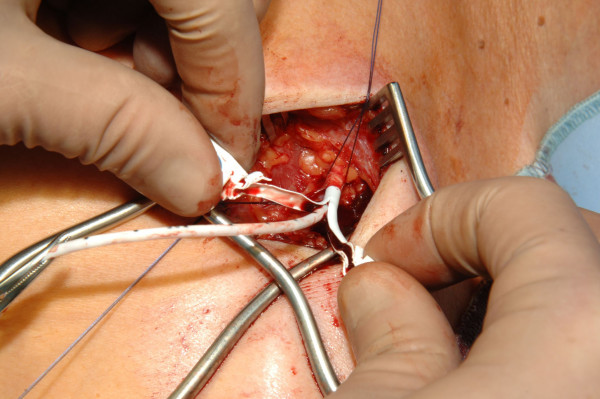
Venae Sectio + Seldinger - Removing of the peel away sheath.

The modified Seldinger Technique is a combination of Venae Sectio and Seldinger Technique using a guide wire and a peel away sheath in the surgically dissected cephalic vein. We believe that the usage of the guide wire and peel away sheath allowes introducting the catheter even in a small cephalic vein and it offers the possiblity to insert the catheter beyond obstacles and narrow curves. The advantage of this technique should be the avoidance of the blind puncture of a central vein (e.g. subclavian vein) and therefore a lower risk of severe complications like pneumothorax, haematothorax and nerve palsy and the possibility to insert the catheter beyond obstacles or narrow curves of the cephalic vein (Fig. 3, 4, 5, 6, 7).

### Medical problem

Correct placement of the TIAP in the superior Vena Cava is mandatory for optimal and save usage of the central venous access. The success rate of TIAP implantation via the conventional approach by transsection of the cephalic vein (Venae Sectio) ranges between 70 to 94%[3]. A multivariate analysis of 400 patients at the Department of Surgery, Universityhospital of Heidelberg, who underwent a primary port-catheter-system engrafting, showed a success rate of the conventional approach of 80%[3]. One of the major causes for failure was an undersized cephalic vein or an obstacle on the way to the superior Vena Cava[3]. Furthermore this study showed that the secondary approach to blind puncture the subclavian vein with Seldinger Technique achieves an overall success rate of 98% but was also characterized by a number of severe complications causally related to indirect puncture, like a pneumo- or hematothorax[1]. In almost all interventional series using the Seldinger technique, pneumothorax as a complication occurred (0 to 3.2%)[3]. In contrast a literature search was not able to identify pneumothorax or nerve palsy after implantation of a TIAP via Venae Section.

## Study design

### Aim of study

The objective of this trial is to compare the success and complication rates of two different implantation techniques for TIAP: Venae Sectio of the cephalic vein and direct insertion of the port catheter versus Venae Sectio and insertion of the port catheter with a guide wire and peel away sheath (modified Seldinger Technique).

### Number of patients needed

Two main factors determine the number of patients needed in a trial. These are the estimated event rate, and the size of the treatment effect.

#### Estimated event rate

The multivariate analysis of 400 patients at our Department who underwent a TIAP implantation showed a success rate of the conventional approach (Venae sectio with insertion of the catheter without a guide wire) of 80%.

#### Size of treatment effect that should be detectable

A modification of the conventional approach as described above must have by surgical decision an expected success proportion of 0.95[1], if surgical practice should be changed.

#### Patients needed

If the real success rate difference is minimum 15% (80% vs. 95%) then there is a 80% chance that a trial involving 160 patients could detect a significant difference at a level of 5%.

### Eligibility

#### Inclusion criteria

• Benign and malignant diseases which demand a safe and permanent venous access, e.g. for chemotherapy and/or parenteral nutrition

• Age equal or greater than 18 years

• Patients scheduled for primary elective implantation of a port-catheter-system in local anaesthesia

• Informed consent

#### Exclusion criteria

• Participation in another intervention-trial with interference of intervention and outcome of this study

• Lack of compliance

• Impaired mental state or language problems (Patient is not able to read german texts)

#### Subject withdrawal criteria

• Randomization will be carried out after preparation of the cephalic vein. If no vein is to exhibit the subject will be removed from the trial and the pre-rand drop-out is to record in the CRF

• At their own request or at request of the legal representative

• If, in the investigator's opinion, continuation of the trial would be detrimental to the subject's well-being

### Consent

Patients who are scheduled for port-catheter-system implantation will have a pre-treatment visit to give the informed consent. During this visit the patient will be screened and informed about the PORTAS trial. In this conversation with the patient the study procedure, risks, benefits and data management will be clarified in detail.

### Randomization and procedures for minimizing bias

#### Minimizing systematic bias

In order to achieve comparable groups for known and unknown risk factors a block randomization will be performed by the randomization software RITA^®^. A sufficient number of patients will be recruited according to the sample seize calculation in order to prevent random error. Patients will get randomized intra operatively after identification of the cephalic vein in order to minimize post randomization drop outs.

#### Minimizing treatment bias

All physicians who participate in this trial will be trained and updated every 3 month to guarantee comparable treatment of patients. Special manuals will be used in operation theatre to reduce random errors. In addition the learning curves of the participating surgeons will be assessed.

#### Minimizing measurement bias

An independent nurse will document and monitor the procedure in the operating theatre. Blinding is not possible and necessary due to the research question.

### Study treatment

All patients will be positioned on the table in a five degree reverse Trendelenburg's position. The neck, chest and shoulders of the patients will be prepared and draped in the customary sterile manner. Antibiotic prophylaxis is only given in patients of risk for endocarditis according to the local standards. Local anaesthesia will be infiltrated in sterile fashion into skin and subcutaneous layer and skin incision will be done 4 cm infer laterally parallel to the clavicle in the deltoid-pectoral region. The cephalic vein is to exhibit. After exhibition of the cephalic vein the randomization will be performed on a Laptop computer system with the RITA^® ^randomization tool.

According to the allocation the procedure will be continued.

#### Intervention-group 1

The cephalic vein will be legated distally and encircled cranially with a 3-0 reabsorbable suture. The vein will be cross-sected ventrally and the catheter flushed with heparinized saline, introduced. Correct positioning will be controlled via fluoroscopy (tip of catheter just at aditus of right atrium) (Fig. 1, 2).

#### Intervention-group 2

The cephalic vein will be ligated distally and encircled cranially with a 3-0 reabsorb able suture. The vein will be cross-sected ventrally and a guide wire will be placed under fluoroscopy to the junction of the superior vena cava and right atrium. After insertion and correct positioning of the wire, a vein dilator and sheath will be passed over the guide wire. The guide wire and dilator will be removed, and the catheter will be introduced through the peel-away sheath. After insertion the peel away sheath will be removed (Fig. 3, 4, 5, 6, 7).

The catheter will be connected to the port chamber. Using the same incision, a subcutaneous pocket will be prepared on the pectoral fascia. The port chamber will be fixed on the fascia of the pectoral muscle with three single non absorbable sutures. Flow for blood withdrawal and infusion is tested via cutaneous puncture (Huber needle). To complete the procedure the system is blocked with isotonic saline.

### Primary and secondary endpoints

#### Primary endpoint

The primary endpoint will be the primary success rate of the randomized implantation technique.

#### Definition of the primary endpoint

Primary success of the randomized implantation technique is defined as the correct position of the catheter checked introperatively by radiography and correct function checked by drawing blood and injecting isotonic saline fluid.

#### Assessment of the primary endpoint

The primary success will be assessed intraoperatively in the CRF. A copy of the intraoperative radiography will be recorded in addition.

#### Secondary endpoints

• Duration of port implantation procedure

• Perioperative complication rate

• Postoperative complication rate

### Safety aspects

#### Specification of safety parameters

#### Training for surgeons

All surgeons will be briefed and trained in both techniques. The correct placement of the port catheter will be checked after operation with x-ray and recorded in the CRF.

#### Concomitant medication

Concomitant medication will not be recorded because an interaction between surgical technique and medication of the patients is unlikely and will therefore not be expected. This decision was met in total agreement of all involved study physicians.

#### Past medical history

Prior and concomitant illness of the patients will be documented in the CRF because discussions between all involved study physicians resulted in the opinion that an interaction between concomitant illness and intervention will be indeed unlikely but could not completely be excluded.

#### Adverse events and serious adverse events

AEs will be reported to the principle investigator in regular intervals throughout the study. Symptoms anticipated by Chemotherapy and malignant illness progress and therefore unlikely related with the surgical implantation technique will not be recorded as AE.

SAEs which are meet one of definitions of the secondary endpoints are treated as SAEs regarding to documentation but have not to be reported to the sponsor/principle investigator within 24 h. They will be reported to the principle investigator in regular intervals throughout the study.

### Analysis

Comparisons will be made of the primary endpoints of both intervention groups for all in the study included patients on an intention to treat basis. Furthermore there will be a second analysis on a "per protocol" basis including only patients who get strictly treated according to the study protocol. The outcome measures of the primary endpoint will be tested for significance with the chi square test.

### Study organization

All patients scheduled for a primary port-catheter-system implantation procedure in the Outpatient-Clinic of the Department of Surgery, University Hospital of Heidelberg, will be referred to the Centre for Clinical Studies in Surgery (KSC) and screened by members of the KSC [4]. The result of the screening will be recorded in the screening-log.

Approximately 500 patients per year undergo a port-catheter-system implantation at the Outpatient-Clinic of the Department of Surgery, University of Heidelberg. The estimated time frame to randomize 180 patients is approximately 18 months.

The independent data management and monitoring will be done by the Coordination Centre for Clinical Trials (KKS) Heidelberg.

The principal investigator has the right to terminate the trial and to remove all trial material from the trial centre at any time in consultation with the Clinical Study Team Leader and the Biometrician. Reasons that may require a termination of the trial include the following:

• The incidence or severity of adverse events in this trial indicates a potential health hazard caused by the study treatment

• It appears that patient's enrolment is unsatisfactory with respect to quality and/or quantity or data recording is severely inaccurate and/or incomplete

• External evidence that makes it necessary to terminate the trial

### Financial support

One half of the costs for this study will be financed by the Surgical Foundation Heidelberg (Stiftung Chirurgie Heidelberg), the other half will be sponsored by Fresenius Kabi AG ^©^. This concept of a half-half financial support will help to guarantee the independency of the final study analysis and its results from commercial interests.

**Table 1:** Flowchart according to CONSORT

	To be assessed for eligibility (n = 280)	
		Total to be excluded (n = 100) refusal to participate (n = 40) not meeting inclusion criteria prior to surgery (n = 20) no cephalic vein present (n = 20) other reasons (n = 20)
	To be randomized (n = 180)	
Experimental Group		Control Group
To be allocated to intervention (n = 90) receive allocated intervention (n = 80) don't receive allocated intervention (n = 10)		To be allocated to intervention (n = 90) receive allocated intervention (n = 80) do not receive allocated intervention (n = 10)
To be analyzed (n = 80)		To be analyzed (n = 80)
